# Skeletal muscle mass and mortality - but what about functional outcome?

**DOI:** 10.1186/cc13729

**Published:** 2014-02-17

**Authors:** Zudin A Puthucheary, Nicholas Hart

**Affiliations:** 1 Institute of Health and Human Performance, University College London, Gower Street, London WC1E 6BT, UK; 2 Division of Respiratory and Critical Care Medicine, University Medicine Cluster, National University Health System, 5 Lower Kent Ridge Road, 119074 Singapore, Singapore; 3 Department of Medicine, Yong Loo Lin School of Medicine, National University of Singapore, 5 Lower Kent Ridge Road, 119074 Singapore, Singapore; 4 NIHR Biomedical Research Centre, Guy’s and St Thomas’ NHS Foundation Trust and King’s College London, Westminster Bridge Road, London SE1 7EH, UK

## Abstract

We have known for over a decade that critical illness survivors suffer from significant functional disability after hospital discharge. Muscle wasting is a major contributor to this disability, occurring early and rapidly during critical illness, with the subsequent weakness associated with delayed weaning and prolonged hospital stay. The scale of this long-term public health issue is concerning for two important reasons: increasing numbers of patients survive critical illness, and this is compounded by the lack of interventions to reduce skeletal muscle wasting to combat the functional disability. In the current issue of *Critical Care*, Weijs and colleagues demonstrate an indirect relationship between skeletal muscle mass on admission to the ICU and mortality. Observational data were obtained from 240 critically ill patients, all of whom received abdominal computer tomography scans for clinical reasons. Skeletal muscle volume was calculated for all visible skeletal muscle at the level of the third lumbar vertebra. In both continuous and categorical regression analysis, lower muscle volume on admission was associated with higher mortality, independent of Acute Physiology and Chronic Health Evaluation II score and gender. Interestingly, no association was observed between mortality and body mass index. These data also demonstrate that more than twice as many critical illness survivors with a low muscle mass on admission, compared to those with preserved muscle mass, were discharged to a nursing home. While this approach is novel and the results support the current clinical view in this area, one must regard these data with caution. Clinically relevant details, such as prior functional status, are not available. Despite these caveats, this study has two main messages. Firstly, muscle mass on admission to the ICU is a predictor of mortality and this physiological biomarker should therefore strongly be considered as an outcome measure in interventional studies. Secondly, low admission muscle mass is associated with increased disability and, in the case of this study, associated with an increased frequency of discharge to nursing homes. Further investigation is required to demonstrate the relationship between muscle mass, functional ability and discharge location.

## 

In the current issue of *Critical Care*, Weijs and colleagues [1] demonstrate an indirect relationship between skeletal muscle mass on admission to the ICU and mortality. We have known for over a decade that critical illness survivors suffer from significant functional disability after hospital discharge [2]. Muscle wasting is a major contributor to this disability, occurring early and rapidly during critical illness [3], with the subsequent weakness associated with delayed weaning and prolonged hospital stay [4]. The scale of this long-term public health issue is concerning for two important reasons: increasing numbers of patients survive critical illness, and this is compounded by the lack of interventions to reduce skeletal muscle wasting to combat the functional disability [5].

Although early exercise-based interventions have shown clinical benefit in terms of functional outcome [6], this effect is by no means universal across different healthcare models [7-9]. A recent trial that focused on the continuum of rehabilitation from admission through to post-hospital discharge failed to demonstrate a functional outcomes benefit [8]. In addition to these disappointing results in terms of rehabilitation delivery, data are currently lacking, based on recent trials [10,11], to guide timing and quantity of nutritional delivery, another important factor in skeletal muscle homeostasis [12]. Indeed, recent translational data suggest that high protein delivery may have an adverse impact on muscle protein synthesis during the first week of critical illness [3].

Weijs and colleagues obtained observational data from 240 critically ill patients, all of whom received abdominal computer tomography scans for clinical reasons, and calculated skeletal muscle volume of all visible skeletal muscles at the level of the third lumbar vertebra. In both continuous and categorical regression analysis, lower muscle volume on admission was associated with higher mortality, which was independent of Acute Physiology and Chronic Health Evaluation (APACHE) II score and gender. Interestingly, no association was observed between mortality and body mass index.

While this approach is novel and the results support the current clinical view in this area, one must regard these data with caution, which the authors also acknowledge. Despite the higher mortality being independent of APACHE II and gender, these rather blunt descriptors of patient demographics do not detail the differing admission diagnoses, with multiple chronic diseases observed in those patients with lower muscle mass; these details are absent from this retrospective study. These data also demonstrated that more than twice as many critical illness survivors with a low muscle mass on admission, compared to those with preserved muscle mass, were discharged to a nursing home. Unfortunately, clinically relevant details such as prior functional status are not available.

Despite these caveats, this study has two main messages. Firstly, muscle mass on admission to the ICU is a predictor of mortality and should therefore strongly be considered as an outcome measure in interventional studies. Secondly, low admission muscle mass is associated with increased disability and, in the case of this study, with an increased frequency of discharge to a nursing home. Further investigation is required to demonstrate the relationship between muscle mass, functional ability and discharge location. However, the unanswered question is whether the change in muscle mass admission or the absolute muscle mass on admission is the most important predictor of outcome. This may in fact be relevant to all hospitalized patients rather than just the critically ill.

Whilst muscle wasting occurs early and rapidly during the first week after admission [3], it may be that baseline muscle mass has a greater influence on subsequent functional ability and, as in this retrospective study, overall mortality. Indeed, low muscle mass has been noted as an independent predictor of mortality in chronic ambulant diseases such as chronic obstructive pulmonary disease, congestive cardiac failure and end-stage renal failure [13]. In the elderly, increased mortality has been observed in patients with age-related muscle loss, so called sarcopenia, and in those considered to be functionally disabled as a result of clinical frailty [14]. Despite this, baseline muscle mass has no relationship with muscle wasting *per se*[3], and there have been no studies adjusting for this, particularly in those focused on functional outcome. Such data are necessary so that we can provide evidence demonstrating the clinical benefit of rehabilitation [7]. For muscle mass on admission to ICU to be used as part of a clinical practice algorithm in prognostication, multicentre observational prospective studies are essential.

## Abbreviations

APACHE: Acute physiology and chronic health evaluation.

## Competing interests

The authors declare that they have no competing interests.
